# Isolation of five rice nonendosperm tissue‐expressed promoters and evaluation of their activities in transgenic rice

**DOI:** 10.1111/pbi.12858

**Published:** 2017-11-28

**Authors:** Hao Li, Juan Li, Rongfang Xu, Ruiying Qin, Fengshun Song, Li Li, Pengcheng Wei, Jianbo Yang

**Affiliations:** ^1^ Key Laboratory of Rice Genetic Breeding of Anhui Province Rice Research Institute Anhui Academy of Agricultural Sciences Hefei China

**Keywords:** *Bacillus thuringiensis*, *GUSplus*, nonendosperm tissue‐expressed promoters, transgenic rice

## Abstract

Using promoters expressed in nonendosperm tissues to activate target genes in specific plant tissues or organs with very limited expression in the endosperm is an attractive approach in crop transgenic engineering. In this article, five putative nonendosperm tissue‐expressed promoters were cloned from the rice genome and designated P_*O*__*s*_ _*NETE*__*1*_, P_*O*__*s*_ _*NETE*__*2*_, P_*O*__*s*_ _*NETE*__*3*_, P_*O*__*s*_ _*NETE*__*4*_ and P_*O*__*s*_ _*NETE*__*5*_. By qualitatively and quantitatively examining *GUSplus* reporter gene expression in transgenic rice plants, P_*O*__*s*_ _*NETE*__*1*_‐P_*O*__*s*_ _*NETE*__*5*_ were all found to be active in the roots, leaves, stems, sheaths and panicles but not in the endosperm of plants at different developmental stages. In addition, P_*O*__*s*_ _*NETE*__*2*_, P_*O*__*s*_ _*NETE*__*4*_ and P_*O*__*s*_ _*NETE*__*5*_ were also inactive in rice embryos. Among these promoters, P_*O*__*s*_ _*NETE*__*4*_ and P_*O*__*s*_ _*NETE*__*5*_ exhibited higher activities in all of the tested tissues, and their activities in stems, leaves, roots and sheaths were higher than or comparable to those of the rice *Actin1* promoter. We also progressively monitored the activities of P_*O*__*s*_ _*NETE*__*1*_‐P_*O*__*s*_ _*NETE*__*5*_ in two generations of single‐copy lines and found that these promoters were stably expressed between generations. Transgenic rice was produced using P_*O*__*s*_ _*NETE*__*4*_ and P_*O*__*s*_ _*NETE*__*5*_ to drive a modified Bt gene, m*C* *ry1Ab*. Bt protein expressed in the tested plants ranged from 1769.4 to 4428.8 ng/g fresh leaves, whereas Bt protein was barely detected in the endosperm. Overall, our study identified five novel nonendosperm tissue‐expressed promoters that might be suitable for rice genetic engineering and might reduce potential social concern regarding the safety of GMO crops.

## Introduction

Rice is extensively planted in many countries and is a critical global food crop (Zhang, 2005). Biotic stresses, such as insect pests, are major challenges to sustainable rice production. Two major rice pests, rice leaf folders and stem borers, cause up to 10% and 30% of the average annual damage, respectively (Krishnaiah *et al*., 2012). To improve insect resistance, a variety of foreign genes have been introduced into rice transgenically (Saha *et al*., 2006; Tu *et al*., 2000). Among them, the *Bacillus thuringiensis* (*Bt*) gene that encodes insect‐specific δ‐endotoxins has emerged as the most effective protection against insects (Chestukhina *et al*., 1982). *Bt* genes act in a dosage‐dependent manner, and constitutive promoters frequently facilitate strong *Bt* gene expression, which further increases insect resistance. Several highly active constitutive promoters, such as the *CaMV35S* (*35S*) promoter, the rice *Actin1* promoter (P_*OsACT*_) and the rice cytochrome *c* gene (*OsCc1*) promoter, have been frequently used in crop transgenic research (Jang *et al*., 2002; McElroy *et al*., 1991; Odell *et al*., 1985). However, the constitutive expression of foreign proteins might have adverse effects on transgenic plants; for example, the constant synthesis of foreign gene products in untargeted tissues usually imposes a metabolic burden on plants, leading to energy waste. The excessive accumulation of exogenous proteins during inappropriate developmental periods or in undesired tissues might have unexpected consequences, including delayed development and abnormal morphology (Ma *et al*., 2009; Xu *et al*., 2006). Besides, Bt protein expressed in edible endosperm might arouse food safety questions regarding Bt insecticidal proteins in transgenic rice, although there is no scientific basis or credible evidence supporting such concerns (Rahman *et al*., 2015). It is therefore desirable to engineer plants that express the foreign toxin gene and accumulate transgenic protein only in target tissues.

Utilizing the promoter strategy, especially with tissue‐specific promoters like nonendosperm expression promoters, can reduce energy waste during growth as well as mitigate the food safety controversy related to insect‐resistant transgenic plants. The current number of available, endogenous, nonendosperm tissue‐expressed promoters is limited. Most of these promoters are derived from green tissue‐expressed genes. RuBisCo (rbcs) is a chloroplast‐localized bifunctional enzyme that catalyses the first major step of carbon fixation in photosynthesis and functions as an oxygenase in photorespiration (Allah *et al*., 2013). *rbcs* genes and their promoters exhibit specific green tissue expression in many crops (Huang and Lin, 2007; Huang *et al*., 1999; Kyozuka *et al*., 1993; Nomura *et al*., 2000). Several previous studies have reported the successful expression of the *Bt* gene specifically in green tissues but not in the endosperm or seeds using the *rbcs* promoter (Manikandan *et al*., 2016; Ye *et al*., 2009). Rice P_*D540*_ is a tissue‐specific promoter that only exhibits activity in green tissues such as the leaf, sheath and young panicle. Cry1Ac protein has not been observed in the endosperm or embryo of transgenic plants expressing P_*D540*_ *::Cry1Ac* (Cai *et al*., 2007). P_*DX1*_ is considered another rice promoter specifically active in green tissues but not in the root, anther or mature seeds. In addition, two *cis*‐elements, GSE1 and GSE2, were identified in P_*DX1*_ as positive regulators for green tissue expression (Ye *et al*., 2012). Using the short fragments and *cis*‐elements of these known nonendosperm‐expressed promoters, several synthetic promoters were assembled that also exhibited green tissue‐specific expression (Wang *et al*., 2015b). In addition, the promoter of maize mesophyll cells‐specific expressed *phosphoenolpyruvate carboxylase* (*PEPC*) gene is also well used. In rice, maize and potato, high levels of Bt were produced exclusively in green tissues under the control of the *PEPC* promoter (Datta *et al*., 1998; Hagh *et al*., 2009; Koziel *et al*., 1993; Qiu *et al*., 2010).

Although several green tissue‐specific promoters have been reported, there are few efficient nonendosperm tissue‐expressed promoters for monocot crops. Moreover, the repeated use of the same promoter for simultaneous expression of multiple foreign proteins has a negative impact on transgene expression and stability (De *et al*., 2000; Peremarti *et al*., 2010). In plant transgenes, a series of promoters with different strengths and temporal patterns provide a more flexible choice for expressing a specific target gene appropriately (Popa *et al*., 2004). To characterize novel nonendosperm tissue‐expressed promoters, we identified new five rice genes expressed in most tissues except for endosperm from a transcriptome database. The spatial and temporal expression pattern and activity of the corresponding promoters were then qualitatively and quantitatively analysed in transgenic rice plants using a modified ß‐glucuronidase gene (*GUSplus*) reporter. We also used two promoters to express a *Bt* gene, *mCry1Ab*, which was codon‐optimized for rice (Song *et al*., 2014), and observed no *Bt* expression in rice endosperm. Therefore, these five promoters provide novel, alternative tissue‐specific promoter resources for rice biotechnology and multigene transformation of crops.

## Results

### Identification of five genes expressed in nonendosperm tissues

**Figure 1:**
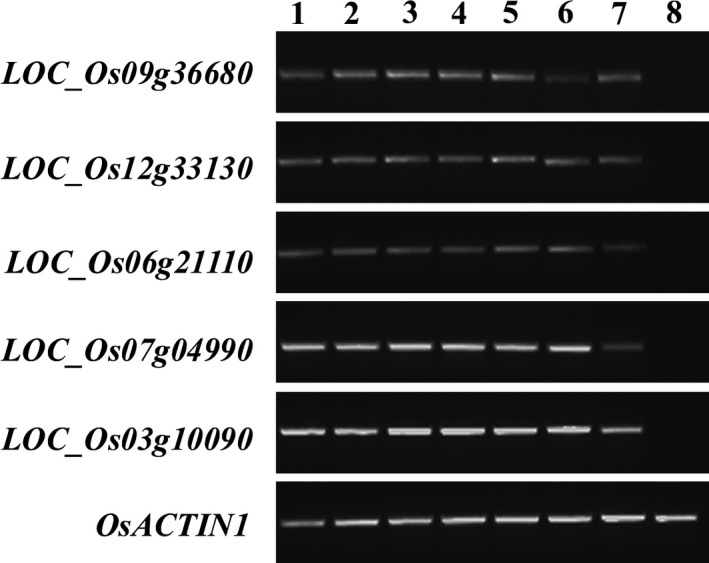
Expression profile of five nonendosperm tissue‐expressed genes. The temporal and spatial expression patterns of five genes were examined by semi‐quantitative RT–PCR using 28‐cycle amplification in different tissues, including the roots (1) and leaves (2) of 10 DAG rice seedlings; the roots (3), leaves (4), stems (5) and sheaths (6) of 60 DAG plants; the panicles of 75 DAG plants (7) and the endosperm of maturing seeds collected at 21 DAF (8). *OsACTIN1* were used to normalize the samples.

To characterize novel rice promoters with strong activity in various nonendosperm tissues but not in the endosperm, five candidate genes with expression in nonendosperm tissues were selected using transcriptome data from the Genevestigator database: *LOC_Os09g36680*,* LOC_Os12g33130*,* LOC_Os06g21110*,* LOC_Os07g04990* and *LOC_Os03g10090*. The expression profiles of these genes are shown in Figure S1. According to the rice genome annotation (http://rice.tigr.org), *LOC_Os09g36680* encodes a putative ribonuclease T2 family domain‐containing protein. *LOC_Os12g33130* and *LOC_Os06g21110* are “expressed protein”. *LOC_Os07g04990* and *LOC_Os03g10090* encode a putative oxidoreductase, aldo/keto reductase family protein and a putative transporter family protein, respectively. The expression patterns of the five candidate genes were then examined using semi‐quantitative RT–PCR. As indicated in Figure 1, *LOC_Os09g36680*,* LOC_Os12g33130*,* LOC_Os06g21110*,* LOC_Os07g04990* and *LOC_Os03g10090* were expressed in roots and leaves, stems, sheaths and panicles but not in the endosperm.

### Analysis of promoter activities in transgenic rice

The regions of 2021, 2018, 2069, 2022 and 1931 bp containing the promoters of *LOC_Os09g36680*,* LOC_Os12g33130*,* LOC_Os06g21110*,* LOC_Os07g04990* and *LOC_Os03g10090* were isolated from the genome of rice variety Nipponbare and designated P_*OsNETE1*_ (nonendosperm tissue expression promoter 1), P_*OsNETE2*_, P_*OsNETE3*_, P_*OsNETE4*_ and P_*OsNETE5*_, respectively. Promoter sequences are supplied in the (Data S1). Each promoter was placed upstream of the visible marker gene *GUSplus*. Meanwhile, as positive controls, a construct harbouring P_*OsACT*_ (2181 bp) and a construct harbouring the previously identified green tissue‐specific expressed *Osrbcs* promoter (P_*Osrbcs*_, 1617 bp) were used (Huang and Lin, 2007; Mcelroy *et al*., 1990). At least thirty independent transgenic rice lines were generated from each construct *via Agrobacterium*‐mediated transformation. The transgene copy number in the T_0_ generation was determined using TaqMan quantitative PCR. After strict self‐pollination, six single‐copy lines with of the T_3_ generation were selected for further analyses.

**Figure 2:**
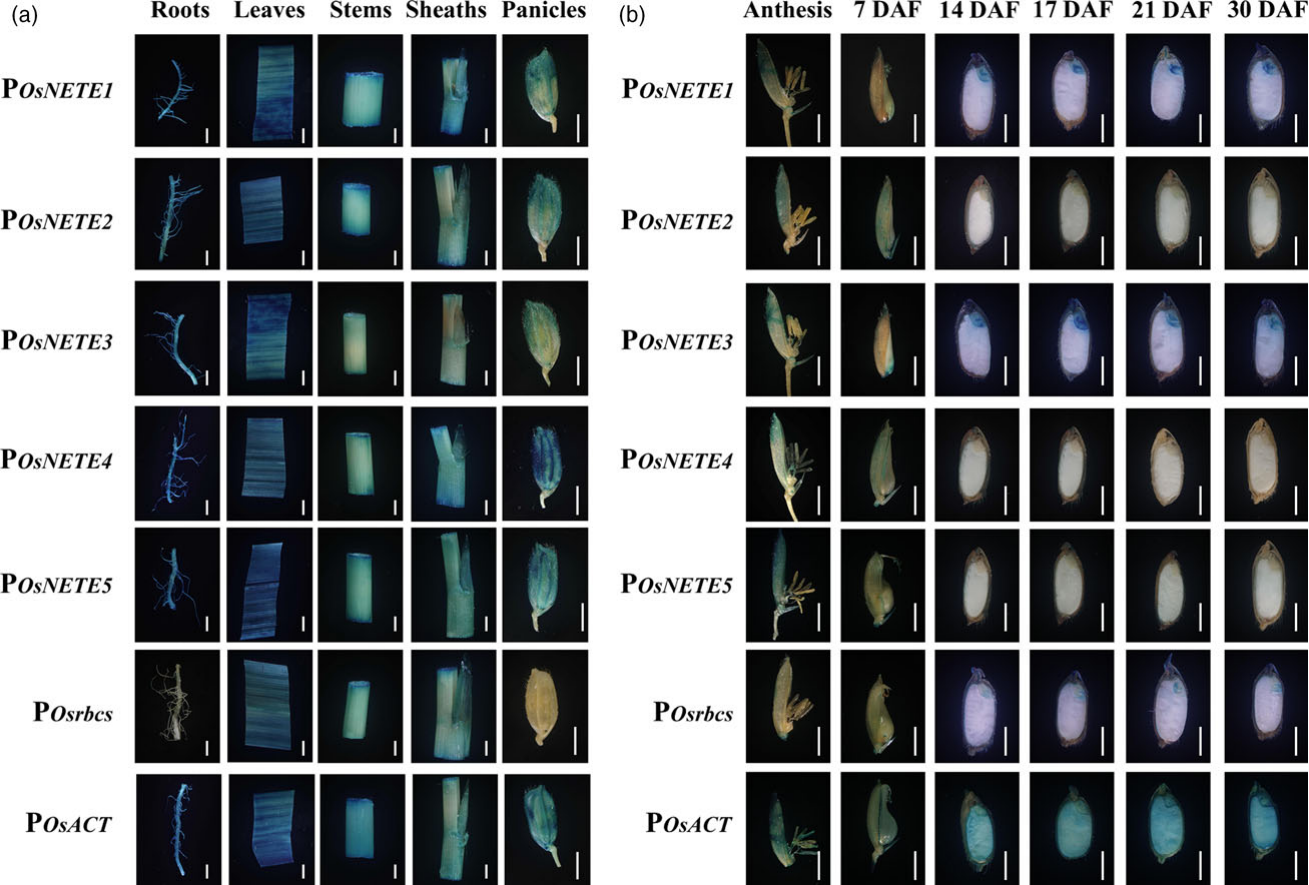
Validation of the tissue specificity of the putative nonendosperm tissue‐expressed promoters in transgenic rice. Histochemical staining was conducted in a series of tissues from transgenic plants including the roots, leaves, stems and sheaths of 60 DAG rice plants and the panicles of 75 DAG plants (a), anthesis and seeds at different developmental stages (b). The seeds were cut into median longitudinal sections. In transgenic plants carrying the construct of the tissue‐expressed promoters, strong blue staining was observed in various nonendosperm tissues after only 2 to 12 h of incubation, while no GUS staining was detected in endosperms after 24 h of X‐gluc incubation. Scale bars represent 2 mm.

In transgenic plants harbouring various promoters, histochemical staining revealed that P_*OsNETE1*_, P_*OsNETE2*_, P_*OsNETE3*_, P_*OsNETE4*_, P_*OsNETE5*_ and P_*Osrbcs*_ had no activity in mature endosperm, whereas P_*OsACT*_ showed activity in all tissues (Figure 2a). P_*OsNETE1*_–P_*OsNETE5*_ showed activity in roots, leaves, stems, sheaths and panicles in a similar manner to P_*OsACT*_. In transgenic plants carrying P_*Osrbcs*_::*GUSplus*, the reporter gene expression was predominantly present in green tissues but was barely detected in nongreen tissues such as roots. Interestingly, P_*OsNETE1*_, P_*OsNETE3*_ and P_*Osrbcs*_ were expressed in embryos. The temporal expression pattern of promoters during seed maturation was also inspected by the histological staining of seed longitudinal sections from each transgenic line (Figure 2b). To monitor promoter activity during endosperm development, GUS staining was performed in the seeds of the early broom stage (4–10 DAF), milky stage (14–15 DAF), soft dough stage (17–18 DAF), hard dough stage (20–23 DAF) and mature stage (>28 DAF) (Joshi *et al*., 2015), and P_*OsNETE1*_–P_*OsNETE5*_‐driven GUS expression was not observed in the endosperm. These results indicated that the five promoters are functional and expressed in a nonendosperm tissue‐specific manner, although their strength needs further verification.

### Quantitative analysis of promoter activity

**Figure 3:**
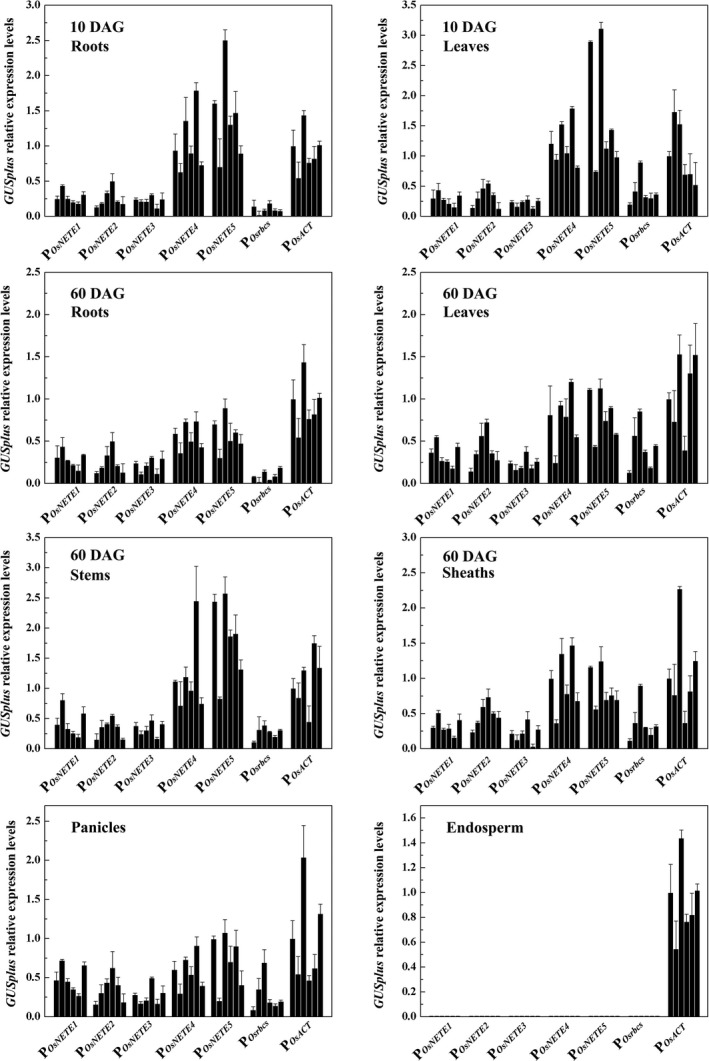
qRT–PCR analysis of the reporter gene expression in six single‐copy transgenic lines harbouring promoter::*GUSplus*. Total RNA was prepared from various tissues including the panicles of 75 DAG plants and the endosperm of maturing seeds (21 DAF). The relative levels of *GUSplus* were measured by qRT–PCR, and *OsACTIN1* was used as the internal control gene. The levels of promoter‐driven reporter gene transcripts were calculated relative to those of *GUSplus* in the P_*O*__*s*_ _*ACT*_ *::GUSplus* transgenic lines. The average was calculated from three biological replicates. ±SD is indicated.

To compare promoter activities, the mRNA levels of *GUSplus* were measured by qRT–PCR in six independent T_3_ lines. As shown in Figure 3, the accumulations of *GUSplus* mRNA in endosperm and nonendosperm tissues were consistent with the histochemical staining results. No significant expression of P_*OsNETE1*_, P_*OsNETE2*_, P_*OsNETE3*_, P_*OsNETE4*_, P_*OsNETE5*_ or P_*Osrbcs*_ was detected in the endosperm. These promoters also showed different expression patterns in different tissues. In the roots and leaves of 10 DAG plants, the average expression levels in P_*OsNETE4*_ *::GUSplus* plants were 1.05‐fold and 1.22‐fold higher than those of P_*OsACT*_::*GUSplus* plants, and those in P_*OsNETE5*_ *::GUSplus* plants were 1.41‐fold and 1.71‐fold higher, respectively. Plants harbouring P_*OsNETE1*_, P_*OsNETE2*_ and P_*OsNETE3*_ showed similar *GUSplus* expression levels in the roots and leaves; these levels were one‐half to one‐quarter lower than those in P_*OsACT*_::*GUSplus* plants. The activity of P_*Osrbcs*_ in the leaves was almost half that of P_*OsACT*_, and much lower expression was observed in the roots, similar to previous reports (Wang *et al*., 2015b).

In 60 DAG plants, P_*OsNETE4*_ and P_*OsNETE5*_ also generated higher expression in the roots, leaves, sheaths, stems and panicles than P_*Osrbcs*_, P_*OsNETE1*_, P_*OsNETE2*_ and P_*OsNETE3*_. In leaves and sheaths, the average *GUSplus* mRNA levels produced by P_*OsNETE4*_ and P_*OsNETE5*_ were comparable with those driven by P_*OsACT*_, whereas the activities of P_*OsNETE4*_ and P_*OsNETE5*_ were 1.19‐fold and 1.82‐fold higher than that of P_*OsACT*_ in stems. In panicle tissues, the activities of P_*OsNETE1*_, P_*OsNETE2*_ and P_*OsNETE3*_ were comparable to that of P_*Osrbcs*_ but much lower than that of P_*OsACT*_. Compared to other promoters, P_*OsNETE3*_ exhibited the lowest activity in nonendosperm tissues.

### Comparing promoter activity in the T_3_ and T_4_ generations

**Figure 4:**
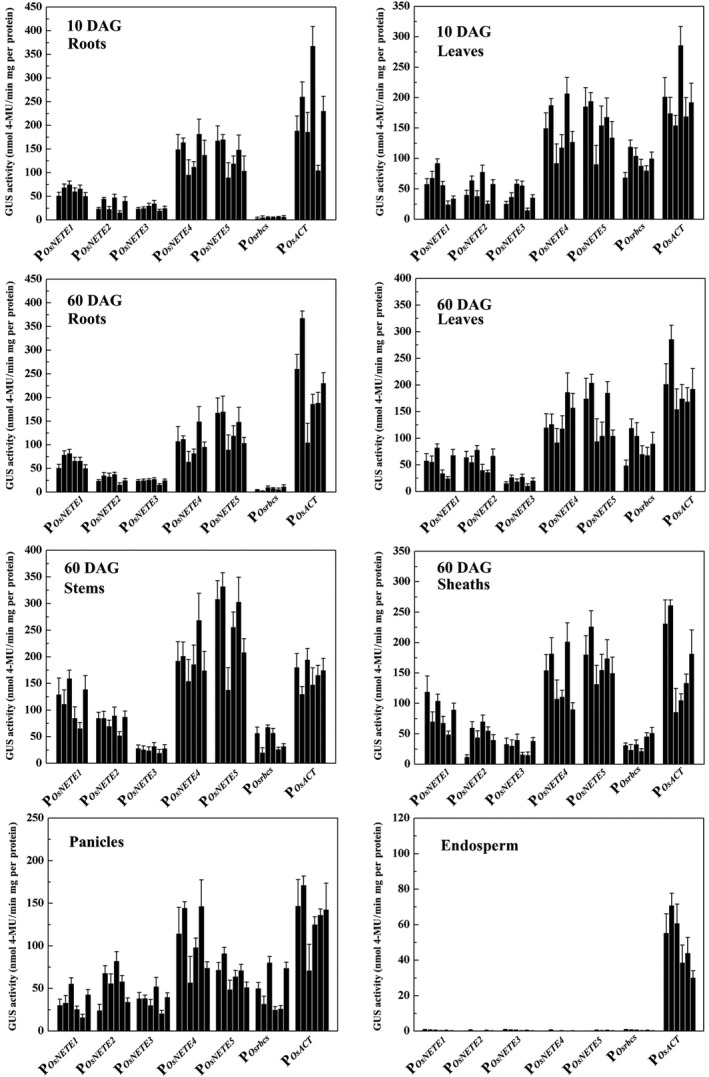
Quantitative analysis of promoter activities in T_3_ lines harbouring promoter::*GUSplus*. GUS activity was measured in the roots and leaves of 10 DAG seedlings; the roots, leaves, stems and sheaths of 60 DAG plants; the panicles of 75 DAG plants and the endosperm of mature seeds. Six independent single‐copy lines from each construct were analysed. GUS activity, given as nmol 4‐methylumbelliferone (MU) per minute per milligram protein, was averaged from three biological replicates. ±SD is indicated.

In plant genetic engineering, an ideal promoter should exhibit a stable expression pattern and strength between different generations. To evaluate generational stability, expression was monitored quantitatively in the T_3_ and T_4_ generations of six single‐copy homozygous lines for each construct using fluorometric GUS assays. The promoter expression pattern was similar to that obtained from the qRT–PCR results in the T_3_ generation, although the relative strength differed slightly among different lines (Figure 4). For instance, very slight background GUS activity from P_*OsNETE1*_‐P_*OsNETE5*_ *::GUSplus* was detected in mature endosperm; P_*OsNETE4*_ and P_*OsNETE5*_ expression levels in roots, leaves, stems and sheaths were higher than those of other nonendosperm tissue‐expressed promoters and were comparable with that of P_*OsACT*_. In P_*OsNETE1*_‐P_*OsNETE5*_ *::GUSplus* plants, the GUS activity ranges were 34.20–115.24, 28.83–78.42, 20.20–38.03, 96.02–196.59 and 75.63–257.91 nmol 4‐MU/min/mg protein in various tissues and at various stages. The promoter activities of the same construct in the T_4_ generation were also assessed and were not significantly different from those in the T_3_ generation (Figure S2), which suggests that the promoters are stably expressed in multiple generations of the transgenic plants.

### Production of transgenic rice plants containing P_*OsNETE4*_ *::mCry1Ab* and P_*OsNETE5*_ *::mCry1Ab*

**Figure 5:**
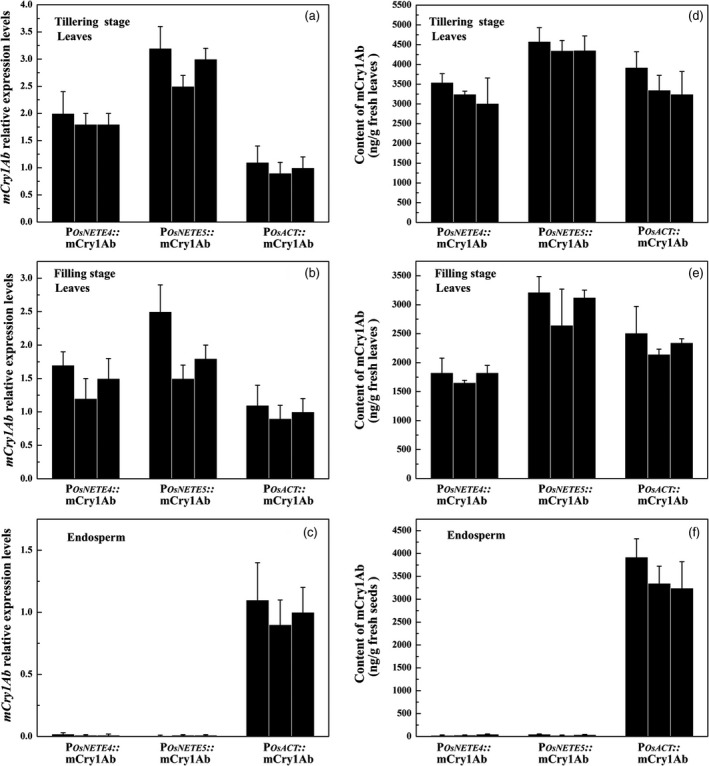
mCry1Ab expression pattern of fresh leaves from three T_2_ homozygous transgenic rice lines. a to c, *mCry1Ab* transcript levels were determined by qRT–PCR in the leaves at the tillering stage (a) and filling stage (b) or in the endosperm of maturing seeds 21 DAF (c) of transgenic plants containing P_*O*__*s*_ _*NETE*__*4*_ *::mCry1Ab*, P_*O*__*s*_ _*NETE*__*5*_ *::mCry1Ab* and P_*O*__*s*_ _*ACT*_ *::mCry1Ab*. The levels of promoter‐driven *mCry1Ab* transcripts were calculated relative to those of *mCry1Ab* in the P_*O*__*s*_ _*ACT*_ *::mCry1Ab* transgenic lines. d to f, mCry1Ab protein levels were measured by ELISA in the leaves at the tillering stage (d) and filling stage (e) or in the endosperm of mature seeds (f). For each construct, three single‐copy transformants were used. Data are the mean ± SD of three independent biological replicates.

To evaluate the application of nonendosperm tissue‐expressed promoters in biotechnology, we chose P_*OsNETE4*_ and P_*OsNETE5*_ to regulate the expression of a modified Bt gene that has been codon‐optimized for rice, *mCry1Ab*, in the rice variety Nipponbare. Twelve T_0_ transgenic lines with a single copy of each construct were obtained. To analyse the Bt protein in later generations, three single‐copy lines were selected, and homozygous plants were generated by strict self‐fertilization. In the T_2_ generation, the *mCry1Ab* transcript levels from endosperm and leaves at the tillering and filling stages were measured by qRT–PCR, and mCry1Ab protein levels were also measured by ELISA. *mCry1Ab* driven by P_*OsNETE4*_ and P_*OsNETE5*_ was highly expressed in leaves but not detected in the endosperm (Figure 5a, b and c). Similar results were observed for mCry1Ab protein levels. At the tillering stage, the mCry1Ab protein levels driven by P_*OsNETE4*_, P_*OsNETE5*_ and P_*OsACT*_ in leaves were 3269.1, 4428.8 and 3506.5 ng/g fresh leaves (Figure 5d), respectively. mCry1Ab expression decreased at the filling stage: 1769.4, 2995.6 and 2333.1 ng/g fresh leaves in P_*OsNETE4*_ *::mCry1Ab*, P_*OsNETE5*_ *::mCry1Ab* and P_*OsACT*_ *::mCry1Ab*, respectively (Figure 5e). However, only very low amounts of mCry1Ab protein were detected in the endosperm (Figure 5f). Therefore, P_*OsNETE4*_ and P_*OsNETE5*_ could minimize Bt protein expression in the endosperm.

## Discussion

Using a specific promoter is one of easiest and most economic strategies to specifically express a foreign gene in particular plant tissue. In rice genetic engineering, the nonendosperm expression of target genes is attractive; therefore, endogenous nonendosperm‐specific promoters are highly desired. In this study, we isolated five tissue‐specific promoters: P_*OsNETE1*_, P_*OsNETE2*_, P_*OsNETE3*_, P_*OsNETE4*_ and P_*OsNETE5*_. These promoters showed strong or moderate strength in green tissues but either no or very slight background activity in the endosperm. These promoters could be used to efficiently express target genes for resistance to pests, such as rice plant weevils, and to diseases (Du *et al*., 2009; Hua *et al*., 2012; Shah *et al*., 2009; Wang *et al*., 2015a). P_*Osrbcs*_ is commonly used to produce insect‐resistant transgenic rice (Qi *et al*., 2013; Tang *et al*., 2006; Yang *et al*., 2014). However, P_*Osrbcs*_ activity was 17% to 29% of P_*OsACT*_ activity in stems, leaves, sheaths and panicles in our study. Similar results indicating that the strength of P_*Osrbcs*_ is not strong enough to drive *Bt* gene in sheaths and stems (Ye *et al*., 2012). To increase Bt levels in chloroplasts, P_*Osrbcs*_ and its transit peptide sequence (tp) were combined to drive the *Bt* gene, which increased the transcript and protein levels by 25‐fold and 100‐fold, respectively (Kim *et al*., 2009). Compared with P_*Osrbcs*_, which is predominantly expressed in green tissue, the five P_*OsNETEs*_ also had relatively higher activity in roots. Some of them may also be used to enhance the biotic stress resistance of underground tissues or to increase the nutrient uptake of root systems. Similar with P_*Osrbcs*_ (Huang and Lin, 2007), P_*OsNETE1*_ and P_*OsNETE3*_ exhibited activity in embryos, indicating that these are nonendosperm tissue‐expressed promoters but not non‐seed‐expressed promoters as well. Because most of the embryo will be stripped during rice processing before cooking, we believe embryo expression will not affect the application of these promoters to *Bt* expression. Among the five nonendosperm tissue‐expressed promoters, P_*OsNETE4*_ and P_*OsNETE5*_ had higher activities in both 10 DAG and 60 DAG plants, especially in stems, sheaths and leaves, which suggests that P_*OsNETE4*_ and P_*OsNETE5*_ may serve as ideal alternatives for genetic engineering. Although P_*OsNETE1*_ and P_*OsNETE2*_ exhibited relatively lower activities than P_*OsNETE4*_, their activities were nonetheless still comparable to or higher than those of P_*Osrbcs*_. Several strategies have been implemented to prevent resistance to Bt, including refugia; high‐dose and low‐dose approaches; multiple‐gene deployment and targeted expression (Bates *et al*., 2005). Expression of Bt toxins at a moderate level allows a proportion of susceptible insects to survive. Although P_*OsNETE3*_ is a moderate nonendosperm promoter that exhibits the lowest activities in leaves, stems, sheaths and panicles, it may also be useful for expressing *Bt* or other target genes for rice transgenic engineering.

Several *cis*‐elements responsible for nonendosperm expression have been identified. The GATACT element is necessary and sufficient for the high and green tissue‐specific expression of the *PNZIP* promoter (Yang *et al*., 2009). Two groups of positive regulatory *cis*‐elements driving green tissue expression, including LPSE1, LPSRE1 and LPSE2, and GSE1 and GSE2, were identified from P_*D540*_ and P_*DX1,*_ respectively (Cai *et al*., 2007; Ye *et al*., 2012). However, the above‐described elements were not observed in the five promoters identified here. On contrary, various light‐responsive elements, such as the I‐box, G‐box, GT‐1 motif, MBS, Sp1 and GATA motif, have been identified in the five promoters (Table S1) and may be involved in light regulation and green tissue‐specific expression (Donald and Cashmore, 1990; Hartmann *et al*., 2005; Lam and Chua, 1990). In addition, we also identified several cis‐regulatory elements involved in endosperm/seed expression, such as the GCN4 motif, Napin motif, RY‐element and Skn‐1 motif (Ericson *et al*., 1991; Ezcurra *et al*., 1999; Takaiwa *et al*., 1991; Wu *et al*., 1998). ABA is an important plant hormone that plays an important role in seed development, mainly during seed dormancy induction, storage products accumulation and desiccation tolerance acquisition. Furthermore, during seed maturation, dehydration for long‐term storage causes severe drought stress. Therefore, ABA‐ and drought‐responsive elements (ABREs and DREs, respectively) often occur in the regulatory regions of genes involved in seeds maturation. Because a few copies of typical ABREs (Mundy *et al*., 1990) and DREs (Dubouzet *et al*., 2003) were found in the five promoters (Table S1), we infer that some silencers or inhibitors exist in the regions of these five promoters that limit the effectiveness of gene expression in endosperm tissue.

The expression of the mCry1Ab protein drastically decreased at the filling stage compared to the tillering stage. Similar results have also been reported in previous studies (Han *et al*., 2008; Manikandan *et al*., 2016; Ye *et al*., 2009). The mechanisms varying Bt protein concentration in plants are rather complicated. Similar to cotton, we speculate that *Bt* overexpression in the earlier stage would lead to post‐transcriptional regulation, resulting in gene silencing and a decrease in Bt protein levels at a later stage (Bakhsh *et al*., 2012). Overall, we obtained five nonendosperm promoters with a variety of strengths and evaluated P_*OsNETE4*_ and P_*OsNETE5*_ activities in Bt rice. We are currently trying to generate transgenic Bt rice using P_*OsNETE1*_, P_*OsNETE2*_ and P_*OsNETE3*_ to evaluate the insect resistance of these plants under field conditions in hopes that the promoters are useful for future rice transgenic breeding.

## Experimental procedures

### Plant material

Japonica Rice cultivar Nipponbare (*Oryza sativa* L.) plants were used for plant transformation and promoter cloning. Rice seeds were sterilized and germinated in pots at 28 °C under a photocycle of 16‐h light/8‐h dark. After growth to three‐leaf stage, rice seedlings were transferred to the field. During the seed maturing stage, samples were harvested at 7 DAF, 14 DAF, 17 DAF, 21 DAF and 30 DAF. To collect the endosperm, one‐quarter of the seed containing the embryo and a small amount of endosperm was cut away with a razor blade. The remaining sample was used as the endosperm in the experiment.

### Promoter cloning and construction of the binary vectors

The approximately 2.0 kb of regulatory sequences upstream of or near the translation initiation site of five rice nonendosperm tissue‐expressed genes was predicted as promoter regions and designated P_*OsNETE1*_, P_*OsNETE2*_, P_*OsNETE3*_, P_*OsNETE4*_ and P_*OsNETE5*_. The promoters were amplified from 50 ng rice genomic DNA by PCR using sequence‐specific primers (Table S2). The cycling conditions consisted of 95 °C for 10 min; followed by 35 cycles of 30 s denaturation at 95 °C, 30 s annealing at 58 °C, and 2 min extension at 72 °C; and a final cycle at 72 °C for 10 min. The strong constitutive promoter P_*OsACT*_ and a previously identified green tissue‐specific promoter P_*Osrbcs*_ were included as positive controls. The putative promoters were cloned and sequenced in a pEASY‐Blunt vector (Transgene, Beijing, China).

A modified binary construct pCAMBIA1391 was used to prepare constructs for rice transformation, in which *GUSplus* replaced *GUS. GUSplus* was PCR amplified from pCAMBIA1305.1, digested with *Nco*I and *Bst*EII and inserted into pCAMBIA1391 at the corresponding enzyme sites. In addition, a mutation in one *Sma*I restriction enzyme site in the adapter sequence between 35S promoter and *HPT* resistance gene of pCAMBIA1391 allowed the *Sma*I site in the multiple cloning sites (MCS) to be used for promoter construction. The promoter fragments were digested from the pEASY‐Blunt vector using the corresponding restriction enzymes and inserted into the modified pCAMBIA1391. The obtained promoter constructs were used for *Agrobacterium*‐mediated transformation.

To create P_*OsNETE4*_::*mCry1Ab*, P_*OsNETE5*_::*mCry1Ab* and P_*OsACT*_::*mCry1Ab* constructs, the *GUSplus* gene in P_*OsNETE4*_::*GUSplus*, P_*OsNETE5*_::*GUSplus* and P_*OsACT*_::*GUSplus* was deleted first. Then, the synthetic, modified *Cry1Ab* gene (*mCry1Ab*) that was codon‐optimized for rice was ligated into the modified vector pCAMBIA1391 under the control of P_*OsNETE4*_, P_*OsNETE5*_ and P_*OsACT*_.

### Transformation of rice and selection of lines for analysis

All of the constructs used for rice transformation were mobilized into the *Agrobacterium tumefaciens* strain EHA105 using a freeze–thaw method. Transgenic rice plants were generated by *Agrobacterium*‐mediated transformation following a previous method (Duan *et al*., 2012). Using a TaqMan PCR assay, single‐copy T‐DNA insertions of T_0_ transgenic plants were screened as described (Yang *et al*., 2005). Six single‐copy T_0_ transgenic plants lines were selected and strictly self‐pollinated. After T_1_ seeds were individually collected, the homozygous plants were identified by segregation based on hygromycin resistance. Then, the T_3_ generation lines generated from a homozygous parent were used for further study.

### RNA isolation and expression analysis

Using the RNAprep Pure Plant Kit (TIANGEN, Beijing, China), total RNA was prepared from plant tissues including the roots and leaves of 10 DAG seedlings; roots, stems, leaves and sheaths of 60 DAG plants; panicles of 75 DAG plants; and the endosperm of maturing seeds (21 DAF). cDNAs were synthesized with the FastQuant RT Kit (TIANGEN). Semi‐quantitative RT–PCR was conducted with EasyTaq PCR SuperMix (TransGen) at 94 °C for 2 min following by 28 cycles of 94 °C for 30 s, 58 °C for 30 s and 72 °C for 30 s extension. The PCR products were run on 2.5% (W/V) agarose gels. *OsACTIN1* was used as a control.

For each cDNA sample, qRT–PCR was performed in triplicate using the SuperReal PreMix Plus (SYBR Green) Kit (TIANGEN) as suggested by the provider. The primers used are shown in Table S2. *OsACTIN1* was used as a reference gene, and normalized relative expression of target genes was evaluated by the ΔΔC_t_ method (Livak and Schmittgen, 2001). The average was calculated from three biological replicates, and the error bars represent “±standard deviation’’ (±SD).

### Histochemical and fluorometric GUS assays

Plant tissues at different growth periods were analysed by GUS staining as described previously (Jefferson *et al*., 1987). The samples were vacuum‐filtered for 15 min and then incubated at 37 °C without light in a GUS reaction mixture. The samples were incubated in ethanol to remove chlorophylls and pigments, and images were taken with a dissecting microscope (Li *et al*., 2017).

The fluorometric GUS assay was performed as previously described (Li *et al*., 2015). Rice plant tissues (100 mg) at different growth period including the endosperm of mature seeds were homogenized in GUS extraction buffer (300 μL), and the resulting supernatants were used to assess GUS activity. GUS activity was quantitatively determined using a Fluoroskan Ascent (Thermo‐Fisher, Waltham, MA, USA) and expressed as nmol 4‐methylumbelliferone (MU) produced per minute per milligram of total soluble protein. For each promoter reporter construct, six independent lines were used. For each independent line, three individual plants were used as biological replicates. The average was calculated from three biological replicates, and the error bars are shown as “±SD’’.

### Quantitative analysis of the mCry1Ab protein by ELISA

To measure the accumulation of mCry1Ab protein in different tissues, the protein was extracted from the leaves at the tillering and filling stages and from the endosperm at the mature stage, respectively. Three single‐copy transformants were used, and three biological replicates were performed. Leaf and endosperm tissue (30 mg) from P_*OsNETE4*_ *::mCry1Ab*, P_*OsNETE5*_ *::mCry1Ab* and P_*OsACT*_ *::mCry1Ab* were ground in liquid nitrogen, dissolved in 500 μL of extraction buffer and centrifuged at 12 000 ***g*** at 4 °C for 10 min to collect the supernatant. The total protein concentration of the supernatant was measured according to the Bradford assay. The mCry1Ab protein content was measured by enzyme‐linked immunosorbent assay (ELISA) using the ELISA QuantiPlate™ kit following standard procedures (EnviroLogix, Portland).

## Conflict of interest

The authors declare no conflict of interest.

## Supporting information


**Figure S1** The tissue‐expression patterns of the five genes examined by the Genevestigator database.
**Figure S2** Quantitative analysis of promoter activities in T_4_ lines harboring promoter::*GUSplus*.
**Table S1** *In silico* analysis of cis‐acting regulatory element in P_*OsNETE1*_, P_*OsNETE2*_, P_*OsNETE3*_, P_*OsNETE4*_ and P_*OsNETE5*_.
**Table S2** Primers used in this study.
**Data S1** Sequences of P_*OsNETE1*_, P_*OsNETE2*_, P_*OsNETE3*_, P_*OsNETE4*_ and P_*OsNETE5*_.
