# Supplementary material for: Isolation of five rice nonendosperm tissue‐expressed promoters and evaluation of their activities in transgenic rice
Source: Plant Biotechnol J. 2017 Nov 28;16(6):1138–47. doi: 10.1111/pbi.12858 (PMC5978396; doi:10.1111/pbi.12858)
Supplement: Supplementary file 1 — Figure S1 The tissue‐expression patterns of the five genes examined by the Genevestigator database. Figure S2 Quantitative analysis of promoter activities in T4 lines harboring promoter::GUSplus. Table S1 In silico analysis of cis‐acting regulatory element in P OsNETE1 , P OsNETE2 , P OsNETE3 , P OsNETE4 and P OsNETE5 . Table S2 Primers used in this study. Data S1 Sequences of P OsNETE1 , P OsNETE2 , P OsNETE3 , P OsNETE4 and P OsNETE5 . [file PBI-16-1138-s001.docx]

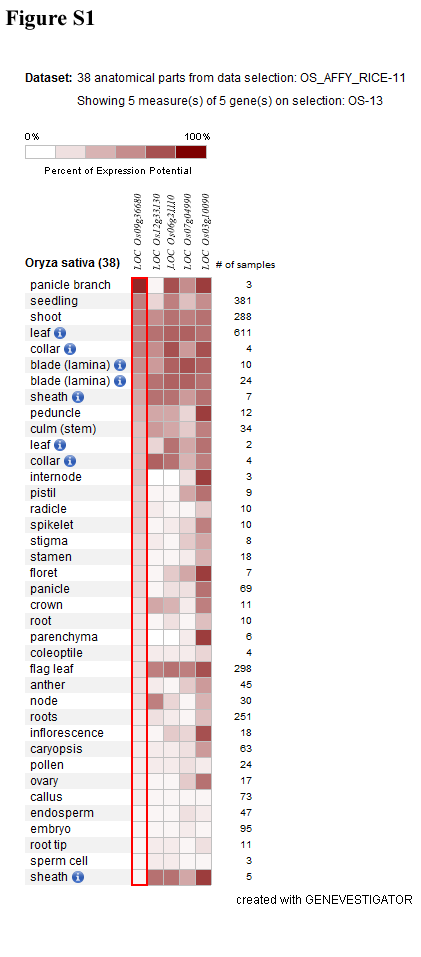


**Supplemental Figure S1.** The tissue-expression patterns of the five genes examined by the Genevestigator database.

The abundance of transcript of genes was examined in 38 individual anatomical parts and was indicated by shade.

**
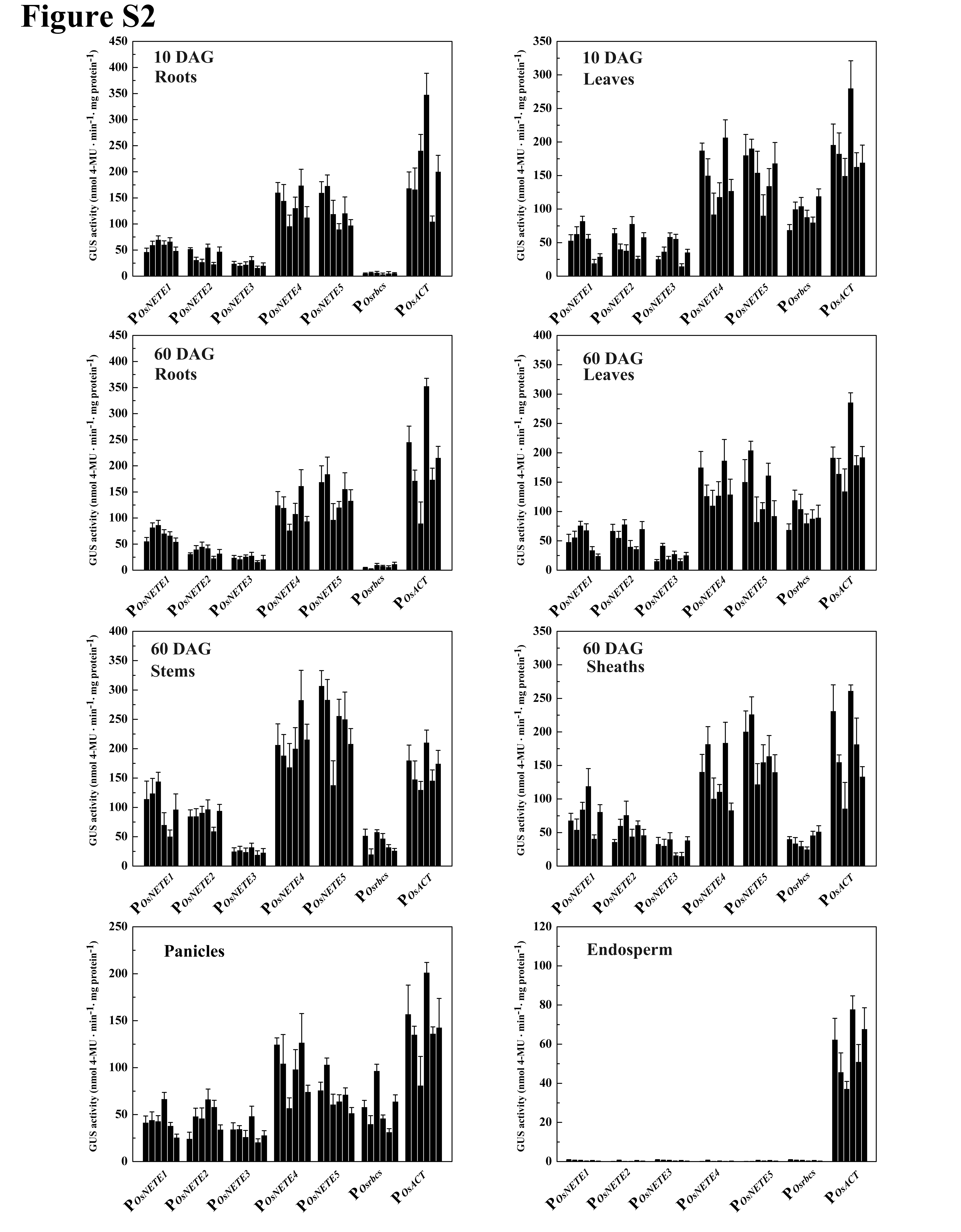
**

**Supplemental Figure S2.** Quantitative analysis of promoter activities in T_4_ lines harboring promoter::*GUSplus*.

GUS activity (nmol 4-MU/min/mg protein) was measured in the roots and leaves of 10 DAG plants; the roots, leaves, stems and sheaths of 60 DAG rice plants; and the panicles of 75 DAG plants and mature endosperm of T_4_ plants. Three biological replicates were performed by fluorometric assay, and the average was calculated. The error bars are given as “± SD”.

**Supplemental Table S1.** *In silico* analysis of cis-acting regulatory element in P*_OsNETE1_*, P*_OsNETE2_*, P*_OsNETE3_*, P*_OsNETE4_* and P*_OsNETE5._*

|  | **Site name** | **Sequence** | **Numbers** | | | | |
| --- | --- | --- | --- | --- | --- | --- | --- |
|  |  |  | P*_OsNETE1_* | P*_OsNETE2_* | P*_OsNETE3_* | P*_OsNETE4_* | P*_OsNETE5_* |
| cis-acting regulatory element involved in light responsiveness and green tissue-specific expression | [*G-Box*](http://bioinformatics.psb.ugent.be/webtools/plantcare/cgi-bin/show_site_info.htpl?QWhere=ID_of_Site%20like%20%27PS~G-Box%27&StartAt=0&NbRecs=10) | ACGT | 3 | 5 | 3 | 5 | 11 |
|  | [*GATA-motif*](http://bioinformatics.psb.ugent.be/webtools/plantcare/cgi-bin/show_site_info.htpl?QWhere=ID_of_Site%20like%20%27PS~GATA-motif%27&StartAt=0&NbRecs=10) | GATAGGA/T | 1 | 0 | 1 | 1 | 0 |
|  | *GT-1 motif* | GRWAAW | 16 | 27 | 16 | 15 | 9 |
|  | [*Sp1*](http://bioinformatics.psb.ugent.be/webtools/plantcare/cgi-bin/show_site_info.htpl?QWhere=ID_of_Site%20like%20%27ZM~Sp1%27&StartAt=0&NbRecs=10) | CC(G/A)CCC | 3 | 2 | 2 | 0 | 5 |
|  | *IBOXCORE* | GATAA | 7 | 9 | 3 | 5 | 1 |
|  | *as-2 motif* | GATAatGATG | 0 | 1 | 0 | 0 | 0 |
|  | *MBS* | CANNTG | 20 | 20 | 28 | 20 | 32 |
| cis-acting regulatory element involved in endosperm/seed expression | *GCN4 motif* | TGTGTCA | 1 | 0 | 0 | 0 | 0 |
|  | *Skn-1* | GTCAT | 1 | 2 | 3 | 4 | 4 |
|  | *RY-element* | CATGCA | 3 | 1 | 1 | 1 | 0 |
|  | *Napin motif* | TACACAT | 1 | 0 | 0 | 0 | 0 |
|  | *SEF4MOTIFGM7S* | RTTTTTR | 4 | 3 | 10 | 4 | 4 |
| ABA-responsive element | *ABRELATERED* | ACGTG | 4 | 1 | 1 | 5 | 8 |
| Drought-responsive element | *DRECCRTCOREAT* | CCGAC | 0 | 3 | 1 | 1 | 1 |

**Supplemental Table S2.** Primers used in this study.

**1. Primers used for semi-quantitative RT-PCR and qRT-PCR assays**

| **Primer** | **Primer sequence (5’ to 3’)** |
| --- | --- |
| *OsACTIN1*-SemiF: | TCAGCAACTGGGATGATATGGAG |
| *OsACTIN1*-SemiR: | GCCGTTGTGGTGAATGAGTAAC |
| *OsNEFE1*-SemiF: | GGCTTACTTGCGACTTCAGG |
| *OsNEFE1*-SemiR: | CGTTCTTTCTGAGCTGGAG |
| *OsNEFE2*-SemiF: | ATGTCTCGCAACGGGAGCAG |
| *OsNEFE2*-SemiR: | ACCTCTGGAGCTGGTGGTGC |
| *OsNEFE3*-SemiF: | ATGGCGTCCAAGACCATGGT |
| *OsNEFE3*-SemiR: | ATGGTGTTTGGGCTTGGTGT |
| *OsNEFE4*-SemiF: | ACCGTCTTGGGTTATCTTCA |
| *OsNEFE4*-SemiR: | AGAACACCTTGGGCTATTGG |
| *OsNEFE5*-SemiF: | GGAAGGAGCTGCTGCTCAAC |
| *OsNEFE5*-SemiR: | GAACGACGCCACGAACGACA |
| GUSplus-qRT-FP | CAACATCCTCGACGATAGCA |
| GUSplus-qRT-RP | GGTCACAACCGAGATGTCCT |
| Actin-qRT-FP | CCTGACGGAGCGTGGTTAC |
| Actin-qRT -RP | CCAGGGCGATGTAGGAAAGC |
| mCry1Ab-qRT-FP | ATCCCGCCGCAGAACA |
| mCry1Ab-qRT-RP | GAGCGGAACATGGACACG |

**2. Primers used to isolate promoters and construct vectors**

| **Primer** | **Primer sequence (5’ to 3’, sequences underlined to show endonuclease recognition site)** | **Restriction site** | **Binary vector** |
| --- | --- | --- | --- |
| GUSPlus-FP | CCATGGATGGTAGATCTGAGGGTAAATT | *Nco*I | 1391-GUSplus |
| GUSPlus-RP | GGTGACCTCACACGTGATGGTGATGGTGA | *Bst*EII |  |
| P*_OsNEFE1_*-FP: | GTCGACTGGCTGATAAGCATAAGGATAA | *Sal*I | 1391-P*_OsNEFE1_*  -GUSplus |
| P*_OsNEFE1_*-RP: | GAATTCAGTACTCTCTAGGCTTGTTTCT | *Eco*RI |  |
| P*_OsNEFE2_*-FP: | AAGCTTGCGGTAATAGAAATGTGGAGGG | *Hind*III | 1391-P*_OsNEFE2_*  -GUSplus |
| P*_OsNEFE2_*-RP: | GAATTCGGTTAATTATGTGTTTAAAGTG | *Eco*RI |  |
| P*_OsNEFE3_*-FP: | GTCGACTCACAATCCCAGTTAGCCCTCA | *Sal*I | 1391-P*_OsNEFE3_*  -GUSplus |
| P*_OsNEFE3_*-RP: | GAATTCGACCACCGTACGTAGGCTTAGC | *Eco*RI |  |
| P*_OsNEFE4_*-FP: | GTCGACAGAGTGGAGAGAATTTGAACCG | *Sal*I | 1391-P*_OsNEFE4_*  -GUSplus |
| P*_OsNEFE4_*-RP: | CCCGGGCCGCCATTGCTCTGCTGCTACG | *Xma*I |  |
| P*_OsNEFE5_*-FP: | AAGCTTGCATTCAAGAAAGCAATCAGCC | *Hind*III | 1391-P*_OsNEFE5_*  -GUSplus |
| P*_OsNEFE5_*-RP: | GAATTCGGCGACCGATCGATTAGCTAGC | *Eco*RI |  |
| P*_OsACT_*-FP: | AAGCTTTGCAGCCCATCCCTCAGCCGCC | *Hind*III | 1391-P*_OsACT_*  -GUSplus |
| P*_OsACT_*-RP: | GTCGACCCGGCGTCAGCCAGCTTCTACC | *Sal*I |  |
| P*_Osrbcs_*-FP: | AAGCTTCGGGTCGAGGTGAACTTTA | *Hind*III | 1391-P*_Osrbcs_*  -GUSplus |
| P*_Osrbcs_*-RP: | GTCGACATGCACTGCTCTGCACAC | *Sal*I |  |
| mCry1Ab-FP | ACTAGTATGGACAACAACCCGAACATCA | SpeI | P*_OsNEFE4_* /P*_OsNEFE5_*/ P*_OsACT_* -mCry1Ab |
| mCry1Ab-RP | GATATCTCAGTACTCGGCCTCGAAGGTC | EcoRV |  |

**3. Primers used for Real-Time PCR assays on the identification of copy numbers**

| SPS-FP | TCTCCTCGTCCAGTGCTTCTC |
| --- | --- |
| SPS-RP | TTGGTGGACGCGCTTCTAG |
| SPS-Probe | TET-TCCTCGCAACCGAAC-TAM |
| HPT-FP | CTATTTCTTTGCCCTCGGACGA |
| HPT-RP | GGACCGATGGCTGTGTAGAAG |
| HPT-Probe | FAM-CGCCGATAGTGGAAACCGACGCCC-TAM |

**Supplemental Data S1:** Sequences of P*_OsNETE1_*, P*_OsNETE2_*, P*_OsNETE3_*, P*_OsNETE4_* and P*_OsNETE5_*

**>P*_OsNETE1_***

TGGCTGATAAGCATAAGGATAAGCGAAAAGATGAGGCCCTAATCTCTCCAGCGGTGTACACTAGACTCTAGATAGGTACCTACTCTGCTAGTGAATGATACGAGCGAATGAACCTTACCAAATTTTGGTAATTTGGCAATATTACCAAATTTTAGCAGTATTTTTTTATGTATTTACGAGAGTTTGGTAAAAAAAACTTCATGGATACACATATTTAGCAATTTTACTTTAAAAAATAGTATGGTTTAAAATGACATTAACCTGAACAACCCATATCTCTTTCAAAAAGCCAACATTAAGAAGTGGTAGCACTAGCATTTTTACCGTTCTTATGAACCGATTTATACAAGGTGAACGACGGGAATCATTATTGTTCTTGTGAGAGAGACACAAGGCAGCTGGGGGACTGGTAGCGAGTAGCTACAGCTTTCTTTTCATTTTTTTTTGAGAATTACACAGTACAACGCAGACACTCATAACGCACGCGTACTCACCCCTATGAACACACGCACGCAAACCCTACCCCTATAAGCTTTTTTTTCAATTTGACTATGTCAGGGAGCATATTTTATGGATATTAAAATGATAGGGCAGAATATACACGTGCATGTATTTCTTAGATTGAATGGGTGGGACCGTATTTCTCCGTGGAGTAAATAGGTGGGGCTGGTCCATCCTGATCGTTTCTGATCGTTAGGTATGGCCGTACGAGTGAAGATAGTTTACTCCAATTTATATACATTCATTGTTCATAAAGTAGCACAATTGAGTAAATTTTGTAAAAACAACTAATACTATTGATTTCCTACAATTTTAAAGGCATATTAGACACTGAAACAATAATAGCCATCACCCATAGATCCGCCGCTCCTGTTGCTGCAGGCAAGGAATCCACCACTACCGTGATTATGGCTGTCACATGAAGCCTATTGTACCATTCCTAAGGCCGTGGCCCTTGGGTCCACCACGTCTCCGTTCTAAGAAAGGGGAGAGGGAAGGGGAAGAGGCAAGGAGCGGGAAAGGAGACGATGATGGCAGGCGGAGCGAATCAGCCCTGGGGGAGGCGGTGTGGAGGGGATGGTGGAGAGGAGAGGAGTGTCAAGGGAAGAAGAGAAAAAGGGGAGGAATAGGATGAGATAAAAAGGTGGAGGTGCGGGGAAAAGGGTTGCAGCCAATGGGGATGCACAGGATGGCTCGTCTAGCTTCATGCAAAATATTAATGGTCCACTAGCTCTTCACGTATATTTTTGTAGACAGGTTCTTTTATAGCCCATTCATGAAAATAAGGGGGTGTGCGAAAATTATTTTTTTACAGTAGCAACAATTATCTAGATTCACCCATCAAACACACATGTTTCATGTTGGGCATACATGTCAAATACACCTAGATGTGTCAACTTCTTATAAAATTGTGACACTAAGTAATAGTAGTTACATGTTATCGTAGCAGTTTAATGACATGTTAGGTTAAAACTGTTGCAAAATGATAATTTTGACTAAATAATTATAATTTTACGAAAATTACTAATCATGATTAGTTTTATGCGTGCCAATTATCTAGCTCTCTGTTATTAGCTATCTATGCATCGATGGTTTGATTGGTTATGGCATTAATTTGATCAATCACCGTGGATGCCTGTTAGGACAAATTTGCCTTGAACTAGACTGTTTACGTTACTACCTTTTCTTTGGGACTCCACATAATTAGATGCATAGTATTTATTAATTTGATATTAAGTTTCTTCTTAAAACTGCCTTCTTTTCACAAAATGTTAACACTAAAAAAACACATACCTCAGGGTACCTCTTTAAGTACCATAAAAATTCTCCATGAATTAACACTCAAAATTGTCATGCATGTTCCCTTGAAAAACCATAAAAAAGATGGAGAAGAATGAAAGGTCCATTCCTTTGCCTCTTGCCCACAACCTGAGCCTATAAATACCCTCACAATGGTTGGGTAGCCAACCAACAAGCAAGAAGAAATTTCTCTCCTTGTAGAAACAAGCCTAGAGAGTACT

**>P*_OsNETE2_***

GCGGTAATAGAAATGTGGAGGGGCATTATTATAAAAAAAATAGGTGCAGAGGGTATAATCGTCAAGCAGTGACTATACCTTTTTTTTCCTTTTTTTTTGGATTTTTTGATGATATATGAGCGATGCTAACGCAATGAATGATCATATATCAATTTAGTCGGGAAAGAACATAAATAACAAAGTAACTCCTCATTATTGTAATAATAAATTATTAATTTACCGCTAATAATACTGGTAGTATAATACGACCAGTAGAGAACTCTGGAGCATCGCTCAATACTTAACGTATGAAACACATGTTGCAACAAAGGTCGACAGAAGTGCCTTTTAAGTGGATTATAAAAATCATGTCACCATTATCATATTTCACTTCCTTAGGAAGATAACTGAGAGAGGTAGACAATTTTTAAAGTATTTTTCTATTATCTCTAAGTATAGGTAGTATAAATTAAACTAGGAAATATAGTAACTTTCTTGAAAAAAAATAGTACTACTACCTCCGAGTACCTACTCAAAAATACCGTGAAATTACAAATTTTCAGAAGAAAAAATTCAATACATAGCTCATAAAACAATGCGGTACAAGAAAAAAAAGCACAAAAAATTTCCGTTTTCGACTAGAAGCACATATGCCACTGATTTTTCTTCGATTCCGTCGTGGTTGCAGTTATGTAGTCGAGTGTTTAGCCATGAGTTGACACTTACAAATACCAATTTGCCCTTGTTCTATCCCTATAGCATAGGGAACTTGGTTGGCTGCTCTAGCTCCTTTTCTTAATTATTAGTTCGGTCGTGCTTGCAAGTTGCGAGGTGATCTCCGAAAGAAAGGGTGATGACGGCGGCTGCTTCTAAGAGCAAGTTTAATAGTCAATGTAATAGCCAATTCATATAATAGTTGCTTACTATAATATTAATACCTTGTCTCACATATCATACACACATTATATCTTGGAGTCTGTGCTACAGTTGGCTACAGATCTATCGCTAGCTGCTCTTATCTCTCCCTTTATCTATTTAAAATATGTTTATATAGCTAGATTATATCCTGCTATTGTATCTGCTCTGAGGCGTCACGCGCCGATCGAGGAAAAGGGCGACGATGGTGGCTGCTTGCGAGGCGCCGCGGGGTGGGGCGACGACGGGTCGACGACGTCCGACAGTGGCGCGCGGCCGACGGCGGTGGCCTCCGTCTCGAACGTACGGCGGCGGCGACGCCACCCGGAGACGAAGGACAAATTAGTGCGAGCATAAATTTATCTACTCTGATCTAAACGACGTCTAGCGTTCGTTTGCTCAAAATAGGAATGGTGTGTTAGGTATCGAAGGAAAATCGATGGCTGGTGTTTCTAGTCGAAAGCGAAAACTTTTTATGCTATTTTTTGGTACTAGGTTATTTTTTATGTTATGGATCAATTTTTCTTTTTCCACAAAGTTGCGGTAGAAATTAACTTATAGAACATCAATGCAAGAGTCTGATGGTAATTCAAAATTAGAATGCTAAATTTATTACCGTGAGACTCTTGCATAAGTCTTAAGTCTTATGCAAGACTTAAGAAGAGATACAAGAAATTTTAAGACTTAAGTTATAATAAGTGTTTAGAAGGATTAAGTGTGTCCACTATGTATGAATGGCAATTGCCTAGTATTGTGCTATAGAAACTAATAAGTTATTATTATAATCTAGTGTGCATGTTACTAAACGAGATCCACAAATAATTAAGACTTCAAGGTACGTGTACGCTAGCTGATCCTAGGAACAAATGCACACACCATTCACTACAAGCATATATATCAAGATCAACATATTCGATCTCCCAAGAAGAAAAAATGAAAGTCATATATGCAATCTTATCCAGGACAAATTCTCTCCACAGTAACGATAGATGAGTTTTTCGCACTATAAAAGCACACACAAATATTACCACCGTGAACTCAGAGAGCCAAGAGCGCCACCACCAAATTAACACACCTACAAACACTCATACAAAAAAAAAAACACTTTAAACACATAATTAACC

**>P*_OsNETE3_***

TCACAATCCCAGTTAGCCCTCACATAGTCATATATTTAGTTCAAAATAAATAAAAATTTAGCACACCCTATTTTAAGCCTAGCCTTTTAATTTTGTAGGCTAGTTAAGCTCTTTACCACCCTTAGCTACGCGATAGCGATCGGGTGTTGGCCTTGGGACTGTACCTCTCGGGCACAGTCCCGGGTGTTGGCTATCCACACCGTAATTTTTGTGATGTTGAAAGCTGACATAGTATTACTGTATAAGTTTCCCATTATCTATACGATGTTATTACTATTTTTATAAGCAACAATGGGTATGTTCAGTTGCTGGTTCGTGGCTGCAGCGCAGCCAGCACCAACAGTGACGCACGCATGCGGCGCAACTGCAGCTGTAGACGATGCAACAGCAGGCTTGCCGAACAGGCCAAATGAAGTTTGACAAAATTTACTACATAATACTCGGTTTGTGATCTTTACTATTGGACAGCTAAAAAAATATAATATGGCATGAGATACCAAAAACCTGGTAATTTATTGATGGTATAAAACTATTATTTTATTATTTTTAACTAAATAGCAAGAGAAACATTTAGAAAATATGGGTTTGCCCCTGTTCAATAGCTTGTTTTGGACAGGATTTTGGGAGAGTCAATGTGTCTTTTTCATGTGGTCCATCTTTCTATTTGGCTCAAATATAATAAACTACTTGTGGATACTCCGTATGTTGTAGCGGAAAATTTAAAAATACTTCAAAGAAAACTTGAAGAAAACAATTTATAAATCGTGTACACGTAATATCTATAAATGGACACTATTTGTTCTATAATAGTTGATGTGAAATTGTAATTCTACATGACACTTAGATGGTTATGTATCAAGCATAAGAGGGATCCAATATCTATTACTCCCTTCGTTCCATATTGTAAGACTTTTTGGTCTTACATAGATTCATCCATTGATAAATGCATATGTTTTATATGTGTCTAGATTTATTACCATCTATATAAATGTGAGTAATGCTAAAAAGTTTTACATTGTAAAACGGAGTTAGTAGTTTTCTATGCACATTAAGCTTTATGTCAATAACTACATTTTGTGGGTTATAAATGCATGAGAGGGATCAAAGGCTATTTTTAGATGATGTGACTTAATGCATATAAAACTTTTGGTCCTAACTATAGAGGATTATAGATTGGATGCAGTATCTCTCAGACTTGGGAAATTGCCAGTTTAGAGGCCGGTATTCTGTAGCAACATTTTGCCACGAAGGTTATACTTAAAAACATAATGTACTAACCATTAAAAAGGAGAGGACATCAGAAACACGCTAGATATATATTTAGGTGGTGTTTGGATTCAGGGACTTAACTTTAGTCCCTATATTTATATACTAATTTAGAGTATTAAATTTAGACTACTTACAAAACTAATTATATAAATGAAAGCTAATTCGCGAGACAAATTTTTTAAGCCTAATTAATCCATAATTATAGAATGTTTACTGTAGCATCACATAGGCTAATCATGGATTAATTAGGCTCAATAGATTCGTGTCGCGAATTAGTCCAAGATTATGGATGGGTTTTTATTAATAGTCTACATTTAATATTTATAATTAGTGTCCAAATATCCGATGTGATAGGAACTTAAAGTTTTAGTCCCATCTAAACAGTTCTTAGTCTTTATTTACCTTGTAAAGTGAGGAGATTAAGATTTTTCCTACCCTACTATTTATATCAGAACAGATACCTACTGACCAGTAAAATAGCTGAGAACAATGCATATTTCCATTCCTCCCAAAGATAATTCAGCTGATGATATTTCCATATCTCAGAGTCTGGTAGGACGTCCTGTGAACAAATTGGAGTGCCAGCTCCAAAAACATGAACATCCATCCTTCGTCGGCATTCCAAAACATCTATTTCTCCTGCCTTCTGCCTGCCGGTCTACACACGGCAATGCAGAGAGCAACGCAAAACGCCAGCACAGTCGCAGTACAAATAAAGCTTTAGCCTTCTCTCTATTAGCACAGCACACTGCTACTAGCTCCATCACAGCTAAGCTAAGCTAAGCCTACGTACGGTGGTC

**>P*_OsNETE4_***

AGAGTGGAGAGAATTTGAACCGGTCAATCATAGACATTATTTCTACTCTTGCACTATATATTATTATTGCGAAGCTTAAATTGAGACATGGCGCATACTTATAGTGAATGCACAGTTTCTTTTTATTTGCTAAGCTTCCATATTGGCTGGACATTTTTAATAAGTAATCCAAATGAAATCATTAGCTTGTAACAATCATGCCCTGTGCGGCTAATGAATTATCCAAAATGATATGTTTCTGTACTTTTCCAGGTTCTATATATGACCCCATGTTTCCTAGATTTAGAATTGCCAGATTCTGTTGAATCAGAATTAAAATTTTTAGCTTATAGCAAGCATTTTCCATATGCTAATAACTTTCTTCCAATGAGATGTTTGGATCCTCTCCAACTATTATATATAATCACTTATTTTACTTTGTACAACAGACCATAAATGGCGATATATACTTGATGGAAGATATGACAGTGCTCCCTGACATTGTGTTCTTGAACTTAATTTTCTTTTCAAGTGGATATTGTATTGGTCCTAGCTTATTCCATGTTCTCAGGATGACCACTGGTGTCAGAAGGTTGAAGCTAGAATTACATAATCATTATAAACGAGAGGTAAAAGCTGAAACTATACTTGGTTTGTTTATCTGATGCAGAGTTACTAAGGAAGTTACGAGAAGCAATTTGTATTTACATTTCCTGTCACACTAGGCTTGATTTGCTATTTTGCTACTTGCCCTACTCTATATAGTGGTTTGATACTGATATATGTAGTAAATGTGTGACATATGCATCTCCTATCGGCAATACTTTGGGGCAACACAACAAGGATTTAGAAGATGATACTAACTGGCTATGCGTTATTGATATTAGAATACTTAGGACGTGCGTAAATTTATACTCCGTTTTCACAGAACTTTCTCATAGGACAGAGGTTGGCTTCAAACTAGTACCCAAGTTGTTATTCTTTTGCAGACTTTTTTATGCTGGTTTATCTCGAGTTGTTCAGGCAGCCTGACAGCTTGAGGCAAGCTTAATGTTGCTACACTAATATACCGCTCTTTTCAGTTTTGTGGATCGGATTGTGTTTGTGATCTTCCACCAAACTGGACATCCGAGGAACTTGTGTTGAACTCCCTTCGTGAAGTGCAAATCACTAACCTGAGAGGAACTGAGAACGAATTTGCTGTTGTGGAGCGGCTATTCAGTTGGGCAGCAGTGCTGAAACAGATGACAATAAATTTCCATAACTCAATCACCGTGAGCACTGCAAGAGAGTTGTGCGAGATGTTACTGAGCTTCTCCAGGCCAGAGATAAGCATGAAATTTTACATTAACCAGGGATCACGTAAGGTTTTGTATGTTCCAGAAGACTAATGTGCCTCATCAGGCTTGCCAGTTGACTAGTGAAGTAGAACTCATTTTGCTTTCTTCCTTTCTCTGATGGATTGATGAAAGAATTCTGTGGACTTGGTTCTTTGATGGTGATGAAACACAACTTATCTGGGTTCCCAATCCTGGAATTGAATGGCAAGAATCAATCTCTTGGTTGGAAAAAAAAATGAAACGAATTTTTTTTTCATCTCATCGTTCTACATGTTGGGTAGGCTTAATGTGTGGCTCCAGCCTCCGGACAGGAAAACTCTTCTGATCTTTCTGCTCAGATATGCTACTGCTGCTAAGCTCAATCTGCATCACACTAGACTGTAGCTGAATGCTACCTGGATTTGTAAGCTAGCCTGGATTTGTATTTCAGCTTGGATTTGTATTTTTGGTCGGATTTATTTGGGCCGTCCATGCAGATATCTCGCAGCAGCAGCAGCATCTGCACGTTACTCACGTGGCATCTCCAACTCTACCGCCAAAATTTGTATTCTTTTTTTGAAAAAAAAATCGTTGGCTCCCGCGACCGCGACGCCTCGTCGCGGCCTTGGTGGTAGAGTACAAGAAGGAGCCAGGCTTCTCTTCTCCTCGAGCCAGCAGCAACACGCACGCACGCACCACACACGTAGCAGCAGAGCAATGGCGG

**>P*_OsNETE5_***

GCATTCAAGAAAGCAATCAGCCGTGATGGTAACAGCCCAAGGCCGCAGCTAGCAGCAAAGCTACGATGCGATCTCCGATGGTACAGGAGAGTTCTTGAACGAACAGTACGTGGCTCTACGGCTAGTCACGCGTTGGGATCGATCCGGCGATGAGATCCGTGTGGGGGCGATCGATGCGAGCAATTTGATCCGATTGATCGCGCTAGCTTTGGTATGGAGTATGGACACAATAAAGGCGGGAAGGTTGAGCTTGAATTGGACGTGCAAGCGTGTGAGTTACTACAACTTACTACGTACTGGAGTACTCCTACAACATTGGGTGGTACGTGGCTCTAGCAACCGGAGCTAGCCGGGGTAGGCGCCTACGGTTAGGTCGAGGCGTGTTGCACGGTCGCAATCTACGTGACGACACGCAGCAGTAGAAAAAAAAAATCTGTAGTACGGTCACGATCGCAAGAGAAACACTGAAGAAACAACAGAAAGACAGAGAATCTATCACATGGTGAAAACCGGTCAGTACGTCGCAGTACAAGTTGTTGTACTCCAGTAATATCGGAAGATAGCTGTCGTCGAACCGGCCAAACTGATAAAAACTAGCGTGATATTTACATCGATCCGCAGCAGAAGAGAGGATATCGATCGATCTAGTGGCGAGAACGAGCTCCGGACGGCGCGCGCTGTGCCAAGATGCGCTTTGTCGACGACGGGTCGGGACCGGGGTGGTGCGGTGCGGTGCGATATGCGAGCGCTCGGTGAATGCGACAGAGATGGTGGCGGCGCGCACGCCGGCCGGCACACACCGCACACGTGAGGATTTTGATGCCATTTGCAAGTTTGCAGGTGGTGCGCGCGCCTGGAGGATGCCATGGTGACGGCGGGTTGACGCATTGGAGAACGCACGCGCGCGCGGCCGATGGCTGCCTGACGGGGGAGGTTTTTGCGTGCGCGCGATGGCCGGAGCGCGTGGTGAGGACCCGGCGGTTGATAGATGCTGCATTGTGTAGTACTCTCTTTGCATTGCAGCTGTACAATTGTGGGAGTGTTTTTACGGTGTACTGGTTTAGACGGTTACGTGTATGCCACTACGTACTTTTTGCCGGTAAAATTGAACTCTGCCGAGTAGCTAAATACAGTCTTTTGGCTTTCAGAAATCTGAGGTGTGTACTGCAGTCTTTGGCTGGAGTACTCTTCCGTATGTTGCACTGCTGTTGATCCCGTGACCGGCCGTATGCCACAGCGCCAGGCTGCCAGCGACAGTTGATACTTGATTGGGTCCTGCCACGACTTGCGTATATACTTGCGGTAACCTGTCAATTGGTCATACGTCAAGGGTCAGTAAACCTCGTGCAAATTGCAGTCTTCCAATGACTTGGAGATACTAGTAAGAAAATTTTAGGAGAGCGAAAAATTAGCTAAACATTTTCTTTCGACAAAGCAAACCCCGTTTTATTTTATTTGTTATAGATCATTGATGTGAGACACCATCATGACCGAAATACAATGTTCCGTAGTAGTCTATCACTCAATCAAAGAGCACTATAACCCACTTTTACAAGTAGACAATACGTTCCTCGCCACACAAAAAAAACTTAATTATGCCGCTTATACCGAACGTGCACTGATTGCCCCCCATTTTCCCTTTTATTTTTTTTCGAACAATCACCAGCAGCCCTGCATATAAAAGAGGGCCAAGGGTTAGCTAGCCACACACCACCTCACTCGTGTTAGCTCGGATCTGTAGCAAAGAGCGAAGAGCGAAGCTGCAGCCTGCAGCTACGCTTGTTCGCTTGAGACCTAGGGTACGAGCAACAATAATAGAGAGTGACTGAGTGAGCGAGCACTGAGTAGCAAGGGCCCGGCACAAACAGGACGCAGCCGGCAACGTGTACGTCGAACCAAACAGAGAACAGCTAGCTAATCGATCGGTCGCC
